# The Impact of Natriuretic Peptides on Heart Development, Homeostasis, and Disease

**DOI:** 10.3390/cells13110931

**Published:** 2024-05-28

**Authors:** Alexandra E. Giovou, Monika M. Gladka, Vincent M. Christoffels

**Affiliations:** Department of Medical Biology, Amsterdam Cardiovascular Sciences, Amsterdam University Medical Centers, 1105AZ Amsterdam, The Netherlands; a.e.giovou@amsterdamumc.nl (A.E.G.); m.m.gladka@amsterdamumc.nl (M.M.G.)

**Keywords:** natriuretic peptides, heart development, cardiac homeostasis, cardiac disease

## Abstract

During mammalian heart development, the clustered genes encoding peptide hormones, Natriuretic Peptide A (*NPPA*; ANP) and B (*NPPB*; BNP), are transcriptionally co-regulated and co-expressed predominately in the atrial and ventricular trabecular cardiomyocytes. After birth, expression of *NPPA* and a natural antisense transcript *NPPA-AS1* becomes restricted to the atrial cardiomyocytes. Both *NPPA* and *NPPB* are induced by cardiac stress and serve as markers for cardiovascular dysfunction or injury. *NPPB* gene products are extensively used as diagnostic and prognostic biomarkers for various cardiovascular disorders. Membrane-localized guanylyl cyclase receptors on many cell types throughout the body mediate the signaling of the natriuretic peptide ligands through the generation of intracellular cGMP, which interacts with and modulates the activity of cGMP-activated kinase and other enzymes and ion channels. The natriuretic peptide system plays a fundamental role in cardio-renal homeostasis, and its potent diuretic and vasodilatory effects provide compensatory mechanisms in cardiac pathophysiological conditions and heart failure. In addition, both peptides, but also CNP, have important intracardiac actions during heart development and homeostasis independent of the systemic functions. Exploration of the intracardiac functions may provide new leads for the therapeutic utility of natriuretic peptide-mediated signaling in heart diseases and rhythm disorders. Here, we review recent insights into the regulation of expression and intracardiac functions of *NPPA* and *NPPB* during heart development, homeostasis, and disease.

## 1. Introduction of Natriuretic Peptides

More than 40 years ago, in 1981, de Bold and colleagues [1] demonstrated that intravenous administration of atrial extracts, but not ventricular extracts, in rats led to increased natriuresis and a decrease in blood pressure, which led to the identification of the atrial natriuretic factor (ANF) or atrial natriuretic peptide (ANP). The term ANP is commonly used and therefore referred to as such in this review. Soon thereafter, the brain natriuretic peptide (BNP) was identified in porcine brain tissue [2], and later studies demonstrated its enrichment in the ventricles of heart failure patients [3,4]. The third member of the natriuretic peptides (NPs), the C-type natriuretic peptide (CNP), was also isolated from the porcine brain and was found to be a smooth-muscle relaxant [5]. All three NPs have remarkable amino acid sequence similarity, with their 17-residue peptides forming a cyclic structure linked at two cysteine residues. However, in contrast to ANP and BNP, CNP lacks the C-terminal extension after the second cysteine residue [5]. All NPs are produced and secreted by cardiomyocytes (CMs), although CNP is predominantly produced by endothelial cells [6,7]. Additionally, the levels of all NPs are found to be increased in heart failure (HF) proportionally to HF severity [8,9,10]. Systemically, NPs have pleiotropic roles ranging from vascular homeostasis to blood pressure regulation, natriuresis, and renin–angiotensin–aldosterone system inhibition and serve as prognostic and diagnostic markers for heart disease onset and progress. These endocrine functions and clinical implications of the NPs have been extensively studied and reviewed [11]. The aim of this review is to describe the intracardiac autocrine/paracrine actions of the NPs within the heart. We summarize and discuss studies and animal models designed to address either the direct function of the NPs or their selective receptor-mediated signaling within the heart (Table 1). 

## 2. Regulation of *NPPA* and *NPPB* Expression during Development and Disease

*NPPA* and *NPPB* are paralogous genes organized in close physical proximity on Ch.1q36 (human) and Ch.4qE2 (mouse) that are derived from an ancestral CNP-3 gene over 500 Myr ago. The *NPPC* gene on Ch.2q24 (human) and Ch.1qC5 (mouse) is derived from an ancestral CNP-4 gene [59]. During development, *Nppa* and *Nppb* are highly expressed in the differentiating atrial and trabecular ventricular chamber myocardium from E8-9 onwards [60]. In contrast, transcripts of both genes remain undetectable in the sinus venosus (including the sinoatrial node; SAN), atrioventricular canal (atrioventricular node; AVN), and outflow tract. After birth, *Nppa* and *Nppb* remain expressed in the heart (*Nppb* is expressed overall at lower levels than *Nppa*), and while *Nppb* remains expressed in both atria and ventricles, the expression of *Nppa* becomes restricted to the atria, with some residual expression in the peripheral ventricular conduction system [60,61]. In conditions of cardiac stress and hypertrophy, the expression of *Nppa* is reactivated and *Nppb* is upregulated in the ventricular CMs [62]. The induction is restricted to the affected ventricular chamber, i.e., left ventricular stress leads to left ventricular induction, and right ventricular stress, such as seen in pulmonary hypertension, leads to right ventricular induction [63]. Moreover, the expression of both *Nppa* and *Nppb* is induced in the border zone CMs after ischemic injury [64]. 

**Figure 1:**
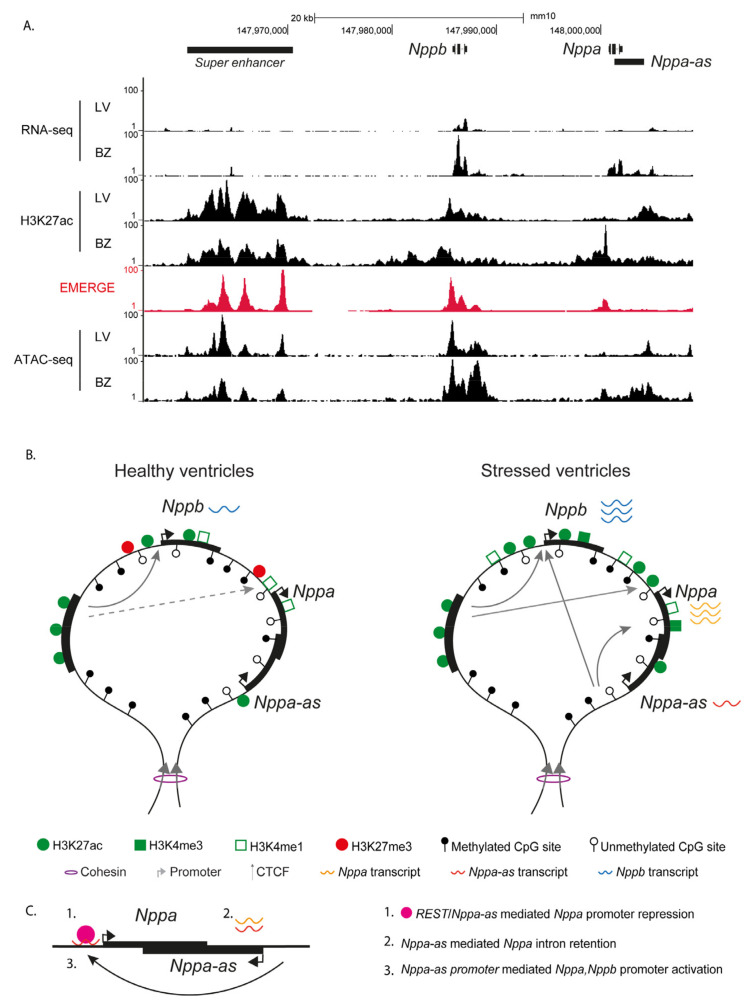
Overview of epigenetic regulation of *Nppa* and *Nppb* (**A**) RNA-seq, H3K27ac, EMERGE, and ATAC-seq in the left ventricle (LV) and the border zone (BZ). (**B**) Overview of epigenetic state changes at the *Nppb, Nppa/Nppa-as* locus in healthy ventricles and left ventricular border zone 7 days after MI (see [64,72] for source data). (**C**) Overview of the proposed mechanisms of *Nppa-as*-mediated *Nppa* regulation.

The proximal promoter of *NPPA* was among the first cardiac-specific promoters to be studied. It was found to be able to drive atrial-specific expression in transgenic mice [65] and has been used as a model to gain insight into the regulation of cardiac gene expression, cardiac transcription factor, chromatin interaction mechanisms, and deciphering the patterning of the atrioventricular canal and chambers during heart development [11,66,67,68]. The control of expression of *Nppa* and *Nppb* during development is regulated by the interplay of distinct developmental and stress-induced regulatory elements that are confined to a CTCF-binding sites-flanked topologically associated domain shared by both genes [69,70]. The proximal promoter sequences of both genes harbor regulatory potential in vivo [65,71]. However, ventricular expression entirely depends on an enhancer cluster (super-enhancer) ≈27 kbp upstream of *Nppa* [72]. Deleting this cluster from the mouse genome resulted in reduced atrial and loss of ventricular expression of both *Nppa* and *Nppb*, both before and after birth. The same study indicated that *Nppa* and *Nppb* compete for activation by the enhancer cluster. However, the response to cardiac injury and induced hypertrophy was still observed in the enhancer cluster-deficient mice, indicating that additional regulatory elements are involved in the stress response [72] (Figure 1). The mechanism underlying the upregulation of *NPPA* and *NPPB* in the ventricles in response to stress or hypertrophy has remained incompletely understood. The promoters of the genes, especially of *NPPA*, in healthy human ventricular CMs are associated with H3K27me3, indicative of polycomb-repressed chromatin after birth [73]. However, in diseased CMs, the genes gain in transcriptionally active histone marks H3K27ac, H3K9ac, and H3K4me3. In this scenario, the always active but very selective super-enhancer can activate its de-repressed target promoters, resulting in strong induction of *NPPA* and *NPPB* [72] (Figure 1). 

The expression of *NPPA* has been shown to be regulated by the natural antisense transcript *NPPA-AS1* in both mouse and human CMs. In both species, the *NPPA-AS1* transcript overlaps with the intron/exon boundaries of *NPPA*. Human *NPPA-AS1* is expressed as multiple alternative spliced isoforms. In vitro studies indicated that *NPPA-AS1* can form duplexes with an intron-retaining *NPPA* transcript, which leads to its downregulation. The intron-retained and alternatively spliced *Nppa* transcripts were undetectable in mice [74] (Figure 1C). Another study described that in human CMs, *NPPA-AS1* acts locally by binding to the *NPPA* promoter, promoting the recruitment of the transcription factor REST. The inhibition of *NPPA-AS1* resulted in upregulation of *NPPA* expression [75] (Figure 1C). Deleting *Nppa-as1* promoted CM proliferation and improved cardiac functionality in both neonatal and adult mice after MI. Mechanistically, *Nppa-as1* was suggested to interact with SFPQ, inhibiting the interaction of the DNA repair complex SPFQ-NOVO in an *Nppa*-independent manner. Additionally, both studies showed that *NPPA-AS1* regulates the *NPPA* expression in the atria but not in the ventricles [75,76]. Epigenetic and transcriptomic analyses in human samples of dilated cardiomyopathy suggested that the promoter of *NPPA-AS1* may act as a trans-regulatory element that is able to interact with the promoters of *NPPA* and *NPPB* and regulate their co-transcription in the diseased heart [77]. 

## 3. Natriuretic Peptide Production and Receptor Interactions

The production, conversion, excretion, processing, and receptor interactions of the NPs have been reviewed extensively [11]. Briefly, *NPPA* and *NPPB* encode the precursors preproANP and preproBNP, respectively, which undergo cleavage and O-glycosylation in atrial CMs. Endoproteolytic proteases corin and furin further process these precursors into biologically active hormones ANP and BNP [78]. CNP also forms precursors, and proCNP is further cleaved by furin [79]. Under physiological conditions, ANP and BNP are produced and secreted mainly by the atria. However, under pathological conditions such as mechanical stress, atrial production and release is increased and ventricular CMs also produce and secrete ANP and BNP [3,4,80,81]. In addition to mechanical stretching, various neurohormonal factors can stimulate the secretion of ANP and BNP [82]. In addition to the circulating BNP and the NT-BNP, failing hearts also secrete proBNP and O-glycosylated-proBNP, the level of glycosylation correlating with the degree of heart failure [83]. In humans, the half-life of ANP in the plasma is approximately 2 min, while that of BNP is significantly longer, reaching 20 min, making it a more effective biomarker for heart failure [3,4,84]. 

NPs exert their physiological actions in multiple tissues through NP receptor-mediated signaling. Three types of NP receptors exist: NP receptor type A; NPR-A (encoded by *NPR1*), NPR-B (also known (encoded by *NPR2*), and NPR-C (encoded by *NPR3*). The genes encoding the receptors are located on Chr.1q21 (human) or Chr.3qF1 (mouse), Chr.9p12 (human) or Chr4.qA5 (mouse) and Chr.5p13 (human) or Chr.15qA1 (mouse), respectively [85]. NP binding to the NPR-A induces the intracellular increase of cGMP, which in turn activates downstream signaling cascades, including cGMP-dependent protein kinases (PKGs), cGMP-gated ion channels, and cGMP-regulated cyclic nucleotide phosphodiesterases (PDEs) [11]. All three receptors are expressed in CMs and cardiac fibroblasts, although NRP-B, under physiological conditions, is not active in the ventricles [86]. Although all NPs can activate the three receptors, their binding affinity varies. ANP and BNP mainly activate NPR-A, while CNP is the primary ligand for NPR-B. NPR-C is characterized as a clearance receptor responsible for the internalization and lysosomal degradation of the NPs. It has the lowest affinity for BNP, which increases its half-life in the circulation [85]. Although NPR-C lacks guanylyl cyclase activity, it has been shown to interact with a Gi protein, thus inhibiting adenylyl cyclase and activating phospholipase C. CNP-mediated activation inhibits L-type calcium currents in atrial and ventricular CMs and pacemaker cells [87]. NPR-C has also been reported to have a non-NP-mediated activity. CNP antagonizes osteocrin for NPR-C binding, thus preventing its degradation and enhancing bone growth during development [88]. An alternative NP clearance pathway involves metalloendopeptidase neprilysin [89], and more recently, BNP has been shown to be hydrolyzed by the fibroblast activation protein (FAP) [90]. 

Deletion of *Npr1* (NPR-A) in mice leads to hypertension, hypertrophy, and cardiac fibrosis [12,40,91,92], whereas genetic ablation of *Npr2* (NPR-B) results in dwarfism and female sterility [93]. *Npr1* knock-out female mice exhibit exaggerated hypertrophy, ventricular dysfunction, and fibrosis during the lactation period, indicating the ANP/BNP–NRP-A system protects against cardiac remodeling during the perinatal period [22]. *Npr2* dominant negative mutant transgenic rats were shown to have increased heart rates and cardiac hypertrophy, while the NPR-A-mediated signaling remained unaffected [94]. In addition, NPR-B has been shown to regulate the automaticity of cardiac pacemaker cells. Mice heterozygous for *Npr2* have impaired sinoatrial node function due to cGMP-mediated ion channel deregulation. However, these mice did not exhibit cardiac hypertrophy or changes in ventricular function [52]. Moreover, it has been described that in the failing heart, the CNP/NPR-B-mediated activity is greatly induced compared to the ANP-dependent activity [86]. NPR-C mutations lead to hypotension and increased bone proliferation, and mice lacking *Npr3* exhibit decreased atrial fibrosis mediated by the inhibition of TGFb1 in the atria [95,96]. Additionally, the deletion of *Npr3* in mice can lead to atrial fibrillation [54,56]. Altogether, these findings indicate that NPRs can differentially regulate the NP-mediated effects by utilizing distinct downstream signaling in a tissue-specific manner and that their preferred activity can alternate under physiological and pathological conditions. 

## 4. The Role of NPs in CM Cell Cycle Activity during Development, Maturation, and Stress

**Figure 2:**
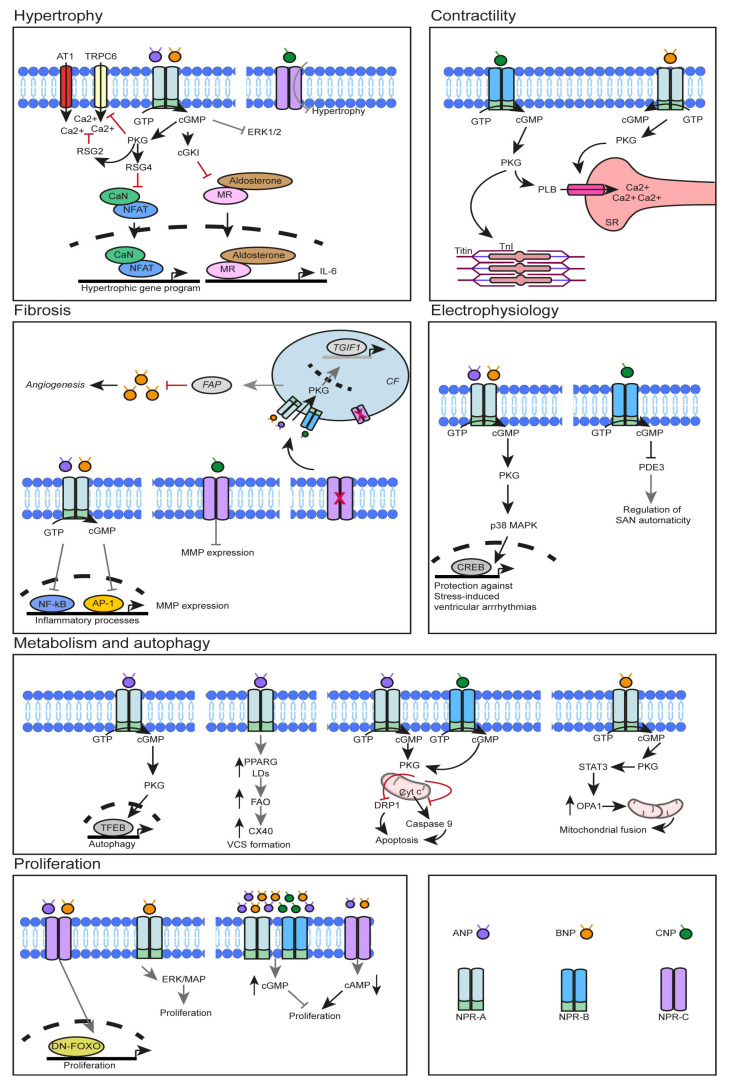
Overview of NP signaling pathways in CMs or involved in crosstalk between cells in the heart. Black arrows; downstream activation, small black arrows; increase or decrease of an effector, grey arrows; underlying mechanistic pathways have not yet been described.

In addition to their cardioprotective functions, NPs have also been implicated in modulating CM cell cycle activity. Neonatal mice with global deletion of *Npr1* have an increased number of CMs, attributed to increased CM proliferation two days after birth (P2). Interestingly, CM-specific deletion of *Npr1* does not impact heart weight and CM cell number, indicating that cardiac hyperplasia is potentially a secondary response to systemic alterations such as arterial hypertension during fetal and neonatal stages [44]. In zebrafish, it was demonstrated that ANP and BNP regulate CM proliferation in a dose-dependent manner and through differential receptor activation. Low levels of the NPs promoted CM proliferation through the NPR-C receptor (Figure 2), while high levels had an anti-proliferative effect mediated via NPR-A and NPR-B. Moreover, in neonatal rat ventricular myocytes (NRVMs), NPR-C mediates proliferation by utilizing its Gi activity, thereby reducing cAMP levels [97]. In cultured embryonic (E11.5) ventricular CMs, the ANP/NPR-A/cGMP axis inhibited CM cell-cycle progression via nuclear p27 inhibition [98]. However, the addition of ANP to cultured chicken embryonic CMs accelerated cell cycle progression by enhancing S-phase entry [99]. Overall, NPs exert complex effects on CM cell cycle activity, depending on factors such as receptor activation, NP levels, cellular context, and species.

NPs have been implicated in proliferation under stress conditions. A recent study investigated the role of BNP in CM proliferation under multiple conditions. Mice with or without MI were intraperitoneally injected bi-daily with BNP. In all conditions, the CM number was increased and accompanied by enhanced expression of markers associated with cell-cycle reactivation. Moreover, the expression levels of cleaved caspase-3, an apoptosis marker, was decreased in the infarct zone (IZ) one day after MI, indicating that BNP has a CM protective role. Interestingly, the administration of BNP to *Npr1*-deficient mice did not increase the number of CMs, suggesting that BNP affects the CM number increase in an NPR-A-dependent manner. In addition, mice subjected to MI were injected with a BNP degradation inhibitor, leading to an increased CM number and cell-cycle reactivation in the infarcted heart. However, the direct involvement of BNP in this condition was not investigated. Lastly, both in vivo and in vitro studies showed that upon BNP administration, the activation of the MAPK/ERK pathway is enhanced, suggesting that this pathway is involved the CM proliferation [46] (Figure 2). ANP and BNP expression was observed to resurge at postnatal day 5 in mice, concomitant with the upregulation of NPR-C expression. Interestingly, in vitro, CMs at P5 appear to undergo a wave of cell cycle reactivation, which generates bi-nucleated CMs rather than more CMs. Stimulation with ANP/BNP and inhibition of FOXO signaling in cultured cells induced cell cycle activation mediated through NPR-C. Intracardiac administration of ANP, together with a dominant negative FOXO mutant, resulted in increased cell cycle activation in P8 mice 24 h after MI [45] (Figure 2).

Upon injury, the border zone (BZ) surrounding the damaged tissue has been implicated as a niche with regenerative capacity [100]. The BZ is marked by strongly induced expression of *Nppa* and *Nppb*. Analysis of the BZ revealed that although the *Nppa/b* expressing area had a transcriptionally and epigenetically different expression profile compared to the CM fetal gene program, there was enrichment of genes associated with dedifferentiated, or immature, CMs. While a direct causal link between the regenerative potential of the BZ and NPs remains unclear, their high expression during cardiac development suggests a potential role for ANP or BNP in fostering a dedifferentiated, pro-proliferative environment in the stressed heart [64]. Taken together, ANP and BNP show distinct effects on CM proliferation during development and in the adult heart under stress conditions. These effects appear to be regulated in a dose-dependent context that involves alternate receptor use.

## 5. The Role of NPs in CM Hypertrophy

The NPs have a well-documented role in attenuating cardiac hypertrophy. During postnatal development, untreated mice lacking *Npr1* develop accelerated hypertrophy and fibrosis between 4 and 12 months of age [13]. Mice with global deletion of *Nppa* exhibit ventricular hypertrophy in a blood pressure-independent manner and under both pressure- and volume overload conditions [28,30,31]. Moreover, *Nppa*^−/+^ and *Nppa*^−/−^ mice exhibit a genotype-dependent degree of hypertrophic and fibrotic response under both normal and pressure-overload conditions, suggesting that ANP participates in cardiac remodeling in a dose-dependent manner [29]. Global deletion of *Nppb* in rats leads to the progressive onset of hypertension, hypertrophic response, and cardiac remodeling, and these phenotypes were prevented by AAV9-mediated BNP delivery [33]. Similarly, long-term overexpression of AAV9-BNP in either normotensive or spontaneously hypertensive rats ameliorated both age-induced and hypertension-induced cardiac remodeling [34]. In addition to pressure/volume overload conditions, *Nppa*-deficient mice also showed cardiac hypertrophy and greater collagen deposition after MI. Untreated *Nppa*^−/−^ mice exhibited smaller infarct sizes and decreased neutrophil infiltration with higher survival rates compared to *Nppa*^−/−^ mice that were chronically infused with ANP [32]. Similarly, two days after 30 min I/R, *Npr1^−/−^* exhibited a smaller infarcted region and lower neutrophilic infiltration than wild type mice [36]. Together, these data highlight the importance of NPs in preventing cardiac hypertrophy and remodeling under different physiological and pathological conditions. 

Under physiological conditions, mice lacking *Nppc* in CMs (cm*Nppc* KO) and fibroblasts (fb*Nppc* KO) showed no adverse alterations in cardiac function or structure. However, upon abdominal aortic constriction (AAC), both cell type-specific *Nppc* KO mice showed a pronounced decline in cardiac function and an increase in fibrosis compared to their wild type counterparts. In contrast, cardiac hypertrophy was observed only in cm*Nppc* KO mice. In the same study, the authors described that the CNP exerts its actions on CMs and fibroblasts in an NPR-C-mediated manner. *Npr3* global deficiency resulted in a significantly worse phenotype of cardiac dysfunction, fibrosis, and hypertrophy upon AAC. Interestingly, subcutaneous infusion of CNP reversed the pathological cardiac remodeling and function in WT mice subjected to AAC, but not in *Npr3*-deficient mice. Furthermore, *Npr3*-deficient and cm*Nppc* KO mice subjected to I/R exhibited a larger infarct size and functional heart recovery. In addition, sympathetic hyperactivation by isoproterenol caused a worsening in cardiac remodeling and function in cm*Nppc* mice. At the same time, fibrosis and hypertrophy were not observed in the global deletion of *Npr2* mice that underwent the same intervention. These findings suggest that the secretion of CNP from CMs and CFs plays a critical role in cardioprotection that is mediated by NPR-C signaling (Figure 2) [35].

Beyond studying the effects of models deficient in the NPs, the studies have also focused on the effects of overexpressing these factors to investigate the effect of their increasing expression levels. Mice overexpressing *Npr1,* specifically in CMs, have a reduced CM cell size either alone or when crossed with *Npr1*-deficient mice [21]. Transgenic overexpression of constitutively active NPR-A specifically in the CMs of mice prevents the hypertrophic response to adrenergic stimulation (isoproterenol) or to pressure overload (AAC) [25]. A different study showed that only male mice with global hyper-activation of NPR-A have smaller hearts due to reduced hypertrophy, and the phenotype is linked with reduced activation of the ERK1/2 pathway [26] (Figure 2). In addition, mice expressing four copies of *Npr1* (*Npr1*^++/++^) had a decrease in heart size when compared to both *Npr1^−/−^* and *Npr1^+/+^* [27]. Together, these data indicate the local involvement of NP-mediated signaling in regulating CM hypertrophy in a dose-dependent manner. 

Multiple studies have shed insights into the mechanistic pathways involved in the regulation of the hypertrophic response by NPs (Figure 2). While CM-specific deletion of *Npr1* in mice does not affect heart size under normal conditions [44], upon stress the heart exhibits pronounced hypertrophy (Figure 2) [20,22,23,24]. Mice with global deletion of *Npr1* showed increased hypertrophy and fibrosis, which were attenuated upon generation of DKO mice together with the angiotensin-II receptor 1. These findings were also observed in the same genotypes that underwent MI, indicating the suppressive role of NPR-A in AngII-mediated cardiac remodeling [16,17]. In mice with *Npr1* CM-specific deletion, it was shown that ANP/cGMP/PKG I signaling is involved in counteracting the hypertrophic response by suppressing the Ang II-stimulated Ca2+ responses by downstream activation of the regulator of G protein signaling (RGS2) [24] (Figure 2). By crossing *Npr1*-deficient mice with mice overexpressing RGS4 in a CM-specific manner, the observed hypertrophy seen in the *Npr1*-deficient mice was reduced by dampening the activation of the pro-hypertrophic calcineurin–NFAT pathway [15] (Figure 2). Similarly, global deletion of *Npr1* also resulted in increased cardiac hypertrophic response, which was attenuated by selective inhibition of TRPC6 activity, a Ca2+ channel, alone or in combination with chronic Ang II treatment [14]. In vitro experiments showed that upon ET-1 stimulation, ANP signaling inhibited the TRPC6-mediated activation of the calcium-dependent phosphatase NFAT (Figure 2). Global deletion of *Npr1* also activates the calcineurin–NFAT pathway, and its selective inhibition attenuates cardiac hypertrophy and fibrosis [18]. Moreover, CM-specific *Npr1* deletion results in increased hypertrophy and adverse cardiac remodeling after TAC, while mineralocorticoid receptor (MR) antagonism alleviates the pathological effects, suggesting that an imbalance between the signaling mediated by these receptors can contribute to pathological remodeling [23]. Female mice with *Npr1* deletion under consecutive lactation cycles exhibit exaggerated cardiac hypertrophy, fibrosis, and cardiac dysfunction. A comparison of multiple tissue-specific *Npr1* deletions revealed that lactation-induced hypertrophy was increased only in CM- or ET-specific *Npr1* deletion and was mediated by an interleukin-6-induced inflammatory response, potentially through excessive aldosterone–MR signaling, indicating that the NP/NPR-A system is involved in the cardioprotection of the maternal heart [22]. During embryonic development, global deletion of *Npr1* at E15.5 results in increased cardiac size with fewer nuclei numbers and reduced levels of Gja1 expression, suggesting that NPR-A signaling is influencing cardiac size and potentially cardiac conduction in the developing heart [12]. Collectively, these findings suggest the multifaceted role of NPs in maintaining cardiac homeostasis by regulating hypertrophic responses and protecting against exaggerated cardiac remodeling under pathophysiological conditions.

## 6. Role of NPs in Cardiac Contractility

A recent study linked CNP-mediated titin phosphorylation to CM stiffness [57]. CNP expression was acutely upregulated in CMs 3 days after TAC, followed by a decline in expression until day 14. Mice with NPR-B CM-specific deletion were subjected to TAC, and at the early stages of pressure overload, CNP prevented CM stiffening by phosphorylating titin (Ser4080) in an NPR-B/cGMP/PKGI-mediated way within the CMs. The cardiac remodeling of the *Npr2^−/−^* hearts was not different compared to WT counterparts after TAC. Based on the CNP expression within different sorted cell types, the authors proposed that endothelial cells act as the primary source of CNP after TAC. Interestingly, mice with *Npr2* CM-specific deletion did not develop any exaggerated TAC-induced hypertrophy and fibrosis compared to their *Npr2^+/+^* counterparts [57] (Figure 2). In an additional study, the authors generated a sensitive FRET-based biosensor system to monitor the levels of cGMP within the CMs compartmentally by targeting either troponin I (TnI) or phospholamban (PLB) in order to determine how NPs regulate cGMP levels intracellularly. The addition of CNP to CMs in vitro resulted in increased levels of cGMP near both TnI and PLB. However, the BNP administration led to cGMP levels only near PLB. Together, these data corroborate the involvement of CNP, but not BNP, in the regulation of CM contractility [58] (Figure 2). 

## 7. Role of NPs in Cardiac Fibrosis and Inflammation

Myocardial fibrosis is a hallmark of heart failure progression and is characterized by altered ECM deposition and the excessive activation of cardiac fibroblasts (CFs), which subsequently can lead to inflammatory responses. Functionally, myocardial fibrosis causes myocardial stiffness and a decline in cardiac output [101]. A critical factor in the establishment of fibrosis is the crosstalk between CMs and CFs [102].

In mouse cardiac fibroblasts, ANP/cGMP/PKG signaling inhibits the TGFb1-mediated translocation of SMAD3 to the nucleus and disrupts their proliferation [103]. Another study suggested that NPR-C might also inhibit CF proliferation in a cGMP-independent manner [104]. However, based on animal studies, ANP and BNP appear to contribute differently to cardiac remodeling. While the deletion of *Nppa* mainly results in an increased hypertrophic response, mice lacking *Nppb* show prominent fibrosis [40,41]. It has been demonstrated that CFs produce and secrete BNP, inhibit collagen synthesis, and upregulate the expression of matrix metalloproteinases (MMPs), important components of ECM degradation, and BNP-mediated suppression of CF proliferation promotes TGF-b, signaling inhibition [105] (Figure 2). Moreover, mice overexpressing BNP exhibited increased neutrophil infiltration and increased MPP expression after MI. However, the mice more frequently died from cardiac rupture compared to the WTs, suggesting that elevated MPP activity can lead to extensive ECM degradation [42]. In addition, intracardiac overexpression of AAV5-BNP has been shown to have context-based effects on cardiac remodeling. BNP delivery during MI improved cardiac functionality, while delivery after chronic AngII administration reduced the fibrotic response [39]. These findings indicate that the levels of BNP might have a critical role in the balance between cardioprotection and pathological remodeling. 

Neonatal, young, and adult mice with global deletion of *Npr1* exhibit a progressive increase in cardiac hypertrophic establishment and fibrosis. While the levels of ANP are unchanged between young and adult mice, BNP levels are upregulated in adult *Npr1*-deficient mice. At all time points observed, there was a noticeable upregulation of MMPs and pro-inflammatory cytokine expression, which was accompanied by increased NF-kB and AP-1 expression and activity. In vivo inhibition of MPP activity attenuated the fibrotic response but did not reduce cardiac hypertrophy [19] (Figure 2). In the following study, the authors investigated the effect of NPR-A in a dose-dependent manner. Mice harboring four copies of *Npr1* (*Npr1*^++/++^) had a markedly reduced size and improvement of cardiac function and exhibited reduced NF-kB/AP1-binding activities and downregulation of pro-inflammatory cytokine expression and the profibrotic cytokine TGF-b1 compared to their WT counterparts (Figure 2). Together, these findings indicate that NP/NPR-A signaling can regulate cardiac remodeling in a dose-dependent manner [27].

A recent study showed that the inhibition of FAP increases vascularization around the infarcted area, promotes ECM deposition, and prevents excessive cardiac fibrosis. In WT mice, inhibition of FAP resulted in cardioprotection and reduced cardiac fibrosis after MI or TAC. Interestingly, these findings were not reproducible in *Nppb*- and *Npr1*-deficient mice, suggesting the critical role of the FAP/BNP/NPR-A pathway in cardiac repair [43] (Figure 2). Moreover, BNP and CNP have been described to play a role, at least in in vitro studies, in reducing inflammation and CF migration by inhibiting the MCP1-mediated pathway that is involved in the monocyte chemotactic movement [106,107].

A recent study identified the role of ANP in promoting autophagy in CMs, both in vitro and in vivo. Specifically, it was shown that ANP induces autophagy through the NPR-A/PRKG pathway and subsequent activation of the master regulator of autophagy-related gene expression, i.e., TFEB (Figure 2). Interestingly, the authors demonstrated that the endogenous secretion of ANP is required for the activation of autophagy under stress conditions in an autocrine/paracrine manner, which exerts cardioprotective effects [38]. Diabetic mice with chronic subcutaneous injection of ANP, BNP or CNP showed decreased fibrosis. However, diabetic mice lacking *Npr3* also showed increased expression of all NPs, accompanied by the most profound reduction in fibrosis. *Npr3* deletion, both in vivo and in vitro, caused increased levels of ANP, BNP, and CNP locally in the heart, which was accompanied by increased intracellular cGMP/PKG levels and unchanged expression of NPR-A and NPR-B. In vitro, exposure of CMs to high glucose upregulated NPR-C expression. Moreover, CFs deficient in *Npr3* activated their cAMP/PKG levels, which were further increased by the addition of the supernatant medium from *Npr3*-deficient CMs, indicating paracrine crosstalk between these two cell types (Figure 2). These findings suggest that NPs act in an autocrine manner to activate their downstream signaling axis in both CMs and CFs. Interestingly, it was shown that CM-derived NPs act in a paracrine manner to further activate the cAMP/PKG pathway [37]. Taken together, NPs appear to have diverse roles in cardiac remodeling and fibrosis by influencing multiple processes in both CMs and CFs.

## 8. NPs in Cardiac Energy Metabolism and Mitochondrial Function

Mitochondria are essential organelles of CMs that play a crucial role in multiple processes of the heart, including ATP production, regulation of Ca2+, and regulation of oxidative stress and cell death [108]. Mitochondrial dysfunction is considered an intricate player in the development of heart failure [109]. Moreover, during development, cardiomyocytes undergo a metabolic shift from glycolysis to oxidative phosphorylation to meet their postnatal growing metabolic requirements, suggesting the involvement of different regulatory players in these processes. Animal studies have established a link between the NP system and the risk of developing obesity and metabolic dysfunction. On a cellular level, ANP has been shown to modulate lipid metabolism in different cell types, including adipocytes and skeletal myocytes, by promoting lipolysis and oxygen consumption [110,111]. Additionally, ANP has been shown to promote epicardial-derived adipogenesis in the adult atria, suggesting that ANP might influence the metabolic activity of these adipocytes and affect lipid metabolism in the heart [112,113]. However, the role of the NPs in autocrine/paracrine CM metabolism during development and disease has not yet been extensively studied. 

During development, ANP expression becomes restricted to the trabecular myocardium, which is the precursor of the ventricular conduction system (VCS), and ANP/NPR-A/cGMP/PKG signaling participates in VCS formation [114]. A recent study investigated the role of ANP in energy metabolism and how this process can influence the establishment and maturation of the VCS in developing CMs. Exogenous ANP stimulation of E11.5 primary ventricular CMs resulted in an increased cell cycle activity of CX40 expressing CMs (Cx40 markers VCS and their precursors), which, however, was not reversible by NPR-A-selective inhibition. Lipidomic and metabolic assays showed that administering ANP can increase lipid uptake and droplet formation within the CX40-expressing CMs but can also drive both glucose and fatty acid utilization to meet their oxidative phosphorylation demands. Moreover, adding ANP or stimulating the adipogenic nuclear receptor PPARG, whose natural ligands are fatty acids, increased CX40 expression and lipid accumulation, with an additive effect when co-treated. These findings suggest that, at least in vitro, ANP, alone or in the presence of activated PPARG, enhances the differentiation of the embryonic VCS by promoting oxidative phosphorylation (Figure 2) [49]. Zebrafish embryos exposed to high-glucose conditions exhibited atrial and ventricular thinning with an overall decrease in CM numbers. Moreover, continuous exposure to glucose resulted in decreased *Nppa* and *Tbx5a* expression, suggesting that modulation of energy metabolism during development can influence morphogenesis and gene expression in CMs [115]. These findings highlight the role of NP/NPR signaling in regulating energy metabolism during CM development and maturation.

In addition to the role of NPs in metabolic regulation of the heart during development, they have also been shown to associate with metabolic changes in the stressed heart. Transcriptomic and proteomic profile analyses of CM-specific *Npr1*-deficient mice showed deregulation of genes involved in metabolic processes and the enrichment of circRNA and microRNA transcripts [47,48]. Single-cell analysis during a TAC time course in mice showed that although the *Nppb* expression levels were similar at different time points after the intervention, the expression levels of *Nppa* among CMs became increasingly heterogeneous independent of the time point. The high *Nppa*-expressing cells had a gene expression profile that differed from that of the low *Nppa*-expressing cells and were enriched in sarcomeric gene transcripts and depleted of transcripts of genes involved in mitochondrial regulation [116]. Another study showed that the silencing of the transcription factor elongation factor A3 (*Tcea3*) in NRVMs after the induction of hypertrophy decreases fatty acid metabolism and promotes oxidative stress while upregulating *Nppa* expression levels [117]. These findings suggest an interplay among NP signaling, energy metabolism, and cardiac remodeling. 

In the well-documented role of cGMP signaling in regulating metabolism, particularly in the cardiovascular system, NPs likely play a role in metabolic stress-induced damage. Indeed, NP-activated cGMP signaling can lead to mitochondria-induced apoptosis by modulating mitochondrial permeability [110] (Figure 2). In cultured CMs, both ANP and BNP have been shown to be protective against hypoxic conditions by preventing the opening of the mitochondrial permeability transition pore opening, in a PI3K/Akt-dependent manner [118,119]. Moreover, in canine CM cultures, administration of ANP suppressed isoproterenol-mediated mitochondrial ROS generation, potentially by decreasing sarcoplasmic reticulum Ca2+ leakage. A recent study explored the direct involvement of ANP and CNP in CM apoptosis. Stimulation of primary CM cultures with ANP and CNP prevented mitochondrial-induced apoptosis by inhibiting caspase 9. By developing sensitive biosensor assays, the authors determined that ANP and CNP increase cGMP levels in the outer mitochondrial membrane, subsequently leading to Drp1 phosphorylation and inhibiting its pro-apoptotic function. Interestingly, compared to ANP, CNP induced a significantly larger overall increase in cGMP levels, both in the cytosol and at the outer mitochondrial membrane (Figure 2). However, ANP resulted in a higher cGMP pool, specifically in the outer mitochondrial membrane of adult CMs, indicating a more targeted effect [50]. In both humans and mice with diabetic cardiomyopathy (DCM), the elevated levels of circulating BNP precede the onset of DCM, and treatment with BNP alleviates DCM in mice. Further analysis, both in vivo and in vitro, showed that administration of BNP protects against mitochondrial oxidative stress and apoptosis by promoting mitochondrial fusion. Under hyperglycemic conditions, BNP acts through NPR-A/PKG to activate the STA3/Opa1 pathway, which subsequently inhibits mitochondrial ROS and promotes mitochondrial fusion [51]. Taken together, these data underscore the importance of the NPs in ameliorating metabolic stress-induced damage of CMs and in protecting against DCM.

## 9. Role of NPs in Cardiac Rhythm

NPs can modulate the electrophysiological properties of the heart, and different mutations, variants (mainly of ANP), and NP aggregates have been implicated in cardiac electrophysiological dysfunction [120,121,122]. The individual roles of ANP and BNP in ventricular arrhythmias were investigated in a recent study [53]. At basal conditions, mice lacking either *Nppa* or *Nppb* exhibited mild ventricular remodeling and increased sudden death after pressure overload (TAC). However, after acute stress induced by isoproterenol infusion, mice deficient in either *Nppa* or *Nppb* developed ventricular arrhythmias both in vivo and ex vivo. Mechanistically, the decreased levels of myocardial cGMP levels led to reduced CREB phosphorylation, and selective CREB inhibition led to stress-induced ventricular arrhythmias in WT mice. Further analysis showed that the dysregulation of the ANP- and BNP-mediated phosphorylation of CREB via cGMP-PKG1-p38MAPK signaling mediates the susceptibility to stress-induced ventricular arrhythmias [53] (Figure 2).

In addition to their effect on ventricular electrophysiology, NPs have been shown to influence the atrial conduction and SAN function in the heart. A recent study characterized the anti-arrhythmogenic role of CNP during myocardial infarction. Perfusion of ex vivo mouse hearts with CNP ameliorated ischemia–reperfusion-induced ventricular arrhythmias. Additionally, administration of isoproterenol in mice blocked the antiarrhythmic effects of CNP, while in vitro, CNP attenuated the isoproterenol-induced arrhythmogenic events. Importantly, the anti-arrhythmogenic effects of CNP were abolished upon PDE2 inhibition or CM-specific *Pde2* deletion [123]. Mice heterozygous for the somatic deletion of *Npr2* (*Npr2*^+/−^) exhibited a reduced heart rate, increased spontaneous sinus pause occurrence, and a prolonged sinus node (SAN) recovery time. Further analysis showed no induction of ventricular remodeling or atrial fibrosis in *Npr2*^+/−^ mice. Optical mapping of isolated pacemaker cells revealed reduced action potential firing frequency and diastolic repolarization accompanied by a reduction of the pacemaker and L-type calcium currents, concomitant with reduced cGMP levels. The reduction of these two currents was reversed and SAN conduction was restored upon restoration of NPR-B/cGMP signaling and downstream inhibition of PDE3 [52] (Figure 2). NPRC is also expressed in the SAN [124]. Mice deficient in *Npr3* exhibit prolonged SAN recovery time and increased atrial fibrillation susceptibility, indicating impaired SAN function. Mice lacking *Npr3* display abnormal atrial fibrosis, leading to increased atrial fibrillation susceptibility and prolonged SAN recovery time. Furthermore, *Npr3*-deficient mice exhibit decreased SAN and atrial conduction velocities due to increased atrial fibrosis rather than ion channel dysfunction, while the ventricular electrophysiological and structural characteristics of the *Npr3*-deficient mice remain unchanged [54]. Angiotensin II administration in WT mice led to increased atrial fibrillation susceptibility with P-wave elongation and decreased atria conduction, as well as SAN dysfunction, which was characterized by a reduced heart rate, prolonged SAN recovery time, and impaired conduction in the right atria. Interestingly, all these conditions were exacerbated in Ang-II-treated NPR-C deficient mice and were accompanied by increased fibrosis in either the atria or the SAN. Selective activation of the NPR-C receptor in WT mice ameliorated the electrophysiological defects of atrial and pacemaker CMs and prevented the fibrotic response [55,56]. Taken together, these findings indicate that the NP–NPR axis plays a crucial role in modulating ventricular and atrial conduction and SAN function by regulating the downstream signaling pathways involving cGMP. Dysfunction or deficiency of the NPs and/or their receptors can lead to ventricular arrhythmias, SAN dysfunction, and atrial fibrillation susceptibility.

## 10. Conclusions

The essential role of NPs in regulating cardiovascular homeostasis is well established. Recent research using genetic animal models and sensitive molecular approaches has revealed the cardiac autocrine/paracrine functions of NPs and has begun to unravel the underlying mechanisms of action within the heart. In addition, it is clear that although the different NPs have cardioprotective effects and counteract common pathogenic stimuli, individual NPs also have unique and distinctive functions and downstream effects based on differential NPR utilization, tissue distribution, processing, half-life, dose, and context. Collectively, the recent research advances have started to reveal the intricate mechanisms by which NPs contribute to cardiac homeostasis by regulating a plethora of pathways. For example, there is increasing evidence for the role of *NPPA-AS* as part of an NP self-regulatory circuit. As an additional example, NPs exhibit unique effects also at the subcellular level where CNP, but not BNP, can specifically influence CM contractility. Interestingly, several studies have demonstrated the growing role of NPs in regulating CM metabolism. In addition to their cardioprotective functions involving autophagy and mitochondria-mediated apoptosis, NPs also appear to influence the patterning of the developing heart. Further studies could elucidate the mechanisms of action of NPs under pathophysiological conditions within the different subtypes of CMs and also investigate how NPs contribute to the different biological processes within these differentially specialized CMs. The utility of NPs as biomarkers for cardiovascular diseases including heart failure and their performance in relation to other biomarkers have been extensively studied (reviewed in [125]). Elucidation of the intracardiac mechanisms of action of the NPs is likely to help us better understand and appreciate their clinical value as biomarkers for the different primary and secondary cardiac diseases.

## Figures and Tables

**Table 1:** Summary of studies that have investigated the intracardiac functions of NPs and NP receptors.

Hypertrophy						
NPs or NP receptors	Experimental model	Manipulation	Age/species	Intracardiac observations	Proposed pathways	Ref.
*Npr1*	Global KO	None	E15.5, ND1	Increased heart size, reduced number of nuclei	N/A	[12]
*Npr1*	Global KO	None	4 months, 12 months/mice	Increased CM size, increased fibrosis	N/A	[13]
*Npr1*	Global KO	TRPC channel inhibition+ Ang II treatment	12 weeks/mice	Reduction in hypertrophy	ANP/cGMP/PKG inhibition of the Ca^2+^ channel TRPC6 activity	[14]
*Npr1*	Global KO	NPRA^−/−^ x RGS4 CM-specific OE	16 weeks/mice	Reduction in hypertrophy	NPRA/RGS4-mediated suppression of the calcineurin–NFAT pathway	[15]
*Npr1*	Global KO	NPRA^−/−^ NPRA^−/−^ x AT1^−/−^ DKO	16 weeks/mice	CM hypertrophy Attenuated hypertrophic response	N/A	[16]
*Npr1*	Global KO	NPRA^−/−^ + MI NPRA^−/−^ x AT1^−/−^ DKO + MI	8–10 weeks/mice	CM hypertrophy Attenuated hypertrophic response	N/A	[17]
*Npr1*	Global KO	Calcineurin inhibitor (FK506)	14 weeks/mice	Reduction in hypertrophy, decreased fibrosis	ANP pathway antagonizes the calcineurin–NFAT pathway to regulate the hypertrophic response	[18]
*Npr1*	Global KO	None	Neonatal (P2), young (4 weeks), adult mice (22 weeks)	Increased CM hypertrophy, increased fibrosis	NF-kB/AP1-mediated MPP activation (fibrosis) disruption of sarcoplasmic reticulum Ca2+ handling (hypertrophy)	[19]
		MMP-inhibition	adult mice (22 weeks)	Increased hypertrophy, decreased fibrosis		
*Npr1*	Global KO	TAC	3–6 months/mice	Cardiac hypertrophy	N/A	[20]
*Npr1*	Global KO	None	8–12 weeks/mice	CM hypertrophy	N/A	[21]
	CM-specific NPRA OE	None		CM cell size reduction		
	Global KO x CM-specific CM NPRA OE	None		Reduced hypertrophic response		
*Npr1*	Global KO	Consecutive pregnancy–lactation cycles	8 weeks/mice	Lactation-induced cardiac hypertrophy	NP/NPRA suppressed aldosterone/MR signaling and cardiac IL-6 expression	[22]
	CM-specific KO					
*Npr1*	CM-specific KO	TAC	8–12 weeks/mice	CM hypertrophy	ANP/cGMP/cGKI-mediated inhibition of MR nuclear translocation	[23]
		TAC+ MR agonist (eplerenone)		Attenuation of CM hypertrophy		
*Npr1*	CM-specific KO	Treatment with AngII/ISO	4–6 weeks/mice	CM hypertrophy	ANP/cGMP/PKG I/RGS2-mediated suppression of Ang II-stimulated Ca2+ handling	[24]
*Npr1*	CM-specific constitutively active NPRA	ISO infusion/AAC	8–12 weeks/mice	Prevention of CM hypertrophy	N/A	[25]
*Npr1*	Global constitutively active NPRA	None	12 weeks/mice	Male-specific CM size reduction	NP/NPRA-mediated reduction of ERK1/2 activity	[26]
*Npr1*	Global *Npr1*^++/++^	None	24–26 weeks/mice	Decreased CM area, reduced inflammatory cytokines,	NPRA/cGMP/mediated suppression of Nf-KB/AP1	[27]
*Nppa*	Global KO	TAC	10 weeks	Increased hypertrophy, increased ECM gene expression	N/A	[28]
*Nppa*	Global KO	*Nppa*^+/−^ + TAC *Nppa*^−/−^ + TAC	9–12 weeks/mice	Dose-dependent cardiac hypertrophy in untreated and TAC mice	N/A	[29]
*Nppa*	Global KO	Aorto-caval fistula (ACF) for volume overload and low-salt diet	8–10 weeks/mice	Increased hypertrophy	N/A	[30]
*Nppa*	Global KO	Low-salt diet	8–9 weeks/mice	Increased CM hypertrophy	N/A	[31]
*Nppa*	Global KO	MI MI + ANP infusion	6–24 weeks/mice	Increased hypertrophy	N/A	[32]
*Nppb*	Global KO	none	4–8 weeks/rats	Progressive hypertrophy, hypertension fibrosis	N/A	[33]
		AAV9-rBNP for 9 months	9 months/rats	Reduced hypertrophy		
BNP	AAV9-rBNP for 9 months	Normotensive	3-4 weeks/rats	Prevention of age-associated hypertrophy and fibrosis	N/A	[34]
		Spontaneously hypertensive rat model		Prevention of hypertension-associated hypertrophy and fibrosis		
CNP/*Npr3*	CM-specific deletion	none	Adult/mice	Preserved cardiac functionality, no increase in hypertrophy or fibrosis	N/A	[35]
		Abdominal aortic constriction		Functional decline, increased hypertrophic response, fibrosis		
		Isoproterenol				
		Angiotensin II		Increased hypertrophic response		
		Angiotensin II + CNP		Decreased hypertrophic response		
		IR		Increased infarct size, prolonged impairment in LV function		
	*Npr3* global deletion	none		Preserved cardiac functionality, no increase in hypertrophy or fibrosis		
		Abdominal aortic constriction		Functional decline, increased hypertrophic response, fibrosis		
		Abdominal aortic constriction + CNP		No reversal of pathologic remodeling		
		IR		Increased infarct size, prolonged impairment in LV function		
Fibrosis						
NPs or NP receptors	Experimental model	Manipulation	Age/species	Intracardiac observations	Proposed pathways	Ref.
*Npr1*	Global KO	Consecutive pregnancy–lactation cycles	8 weeks/mice	Lactation-induced cardiac hypertrophy+ fibrosis	NP/NPRA suppressed aldosterone/MR signaling and cardiac IL-6 expression	[22]
	CM-specific KO					
*Npr1*	Global KO	None	Neonatal (P2), young (4 weeks), adult mice (22 weeks)	Increased CM hypertrophy, increased fibrosis	NF-kB/AP-1-mediated MPP activation (fibrosis) disruption of sarcoplasmic reticulum Ca2+ handling (hypertrophy)	[19]
		MMP-inhibition	Adult mice (22 weeks)	Increased hypertrophy, decreased fibrosis		
*Npr1*	Global KO	30 min I/R	10–14 weeks/mice	N/A	NPRA-mediated suppression of NF-kB-mediated inflammatory processes	[36]
*Npr1*	Global *Npr1*++/++	None	24–26 weeks/mice	Decreased CM area, reduced inflammatory cytokines,	NPRA/cGMP-mediated suppression of Nf-KB/AP1	[27]
*Npr3*	Global KO	*Npr3* ^−/−^	20 weeks/mice	No effect on left ventricular and left atrial fibrosis	NPRC deletion results in cAMP/PKA- and cGMP/PKG-mediated TGIF1 upregulation	[37]
		*Npr3*^−/−^ with STZ-induced diabetic cardiomyopathy		Attenuation of diabetes-induced cardiac fibrosis, no difference in atrial fibrosis		
	AAV9-shNPRC	AAV9-shNPRC with STZ-induced diabetic cardiomyopathy		Attenuation of diabetes-induced cardiac fibrosis		
*Nppa*	Global KO	*Nppa*^+/−^ + TAC *Nppa*^−/−^ + TAC	9–12 weeks/mice	Dose-dependent cardiac hypertrophy and fibrosis both in untreated and TAC mice	N/A	[29]
*Nppa*	Global KO	*Nppa*^−/−^ + I/R	8–10 weeks/mice	Increased infarct size and reduced autophagy	ANP/NPR1/PRKG-mediated stimulation of autophagy through activation of TFEB	[38]
BNP	hAAV5-BNP intracardiac injection	Unmanipulated	rats	Reduced fibrosis, increase capillary density	N/A	[39]
		MI		Improved cardiac function	Normalization of SERCA2 expression and PLN phosphorylation	
		Ang II infusion		Decreased fibrosis	N/A	
*Nppb*	Global KO	None	20 weeks/mice	Increased fibrosis	N/A	[40]
		AAC				
*Nppb*	Global KO	None	20 weeks/mice	Increased fibrosis, Increased sarcomere contraction and disorganized myofibrils	N/A	[41]
		AAC				
*Nppb*	Liver-specific human serum amyloid P component promoter-mediated BNP plasma OE	MI	8–12 weeks/mice	Increased neutrophile infiltration in the infracted area and increased cardiac MMP-9 expression	N/A	[42]
*Nppb*, *Npr1*	Global KO *Nppb* Global KO *Npr1*	TAC + FAP inhibition	8–10 weeks	Decreased fibrosis, improved cardiac function, increased angiogenesis in BNP+/+ and NPRA+/+	Fap inhibition results in BNP/NPRA-mediated cardioprotection	[43]
		MI + FAP inhibition				
Proliferation						
NPs or NP receptors	Experimental model	Manipulation	Age/species	Intracardiac observations	Proposed pathways	Ref.
*Npr1*	Global KO	None	P2/mice	Increase in CM number	N/A	[44]
	CM-specific KO	None		No difference		
ANP	None	Intracardiac ANP + MI	Neonatal (ND7)/mice	No difference observed compared to control littermates	ANP, BNP/NPRC signaling and FOXO nuclear activity cooperatively regulate CM cell cycle activity	[45]
		Intracardiac ANP + DN-FOXO + MI		Reactivation of CM cell cycle, reduced scar formation		
BNP	None	Bidaily BNP IP injection	Neonatal mice	Increased number of CMs, increase in CM cell cycle re-entry	BNP activation of the MAPK/ERK pathway	[46]
	None	Bidaily BNP IP injection+MI	8 weeks/mice	Increased number of CMs, increase in CM cell cycle re-entry, decreased apoptosis		
	Myh6 MerCreMer (tamoxifen -D14)	Bidaily BNP IP injection+MI		Increase in TNTI+/GFP+ cells, increase in CM cell cycle re-entry		
	*Npr1* ^−/−^	Bidaily BNP IP injection		Unchanged number of CMs		
	None	Inhibitor of BNP degradation (LCZ696) + MI		Increased number of CMs, increase in CM cell cycle re-entry		
Metabolism and autophagy						
NPs or NP receptors	Experimental model	Manipulation	Age/species	Intracardiac observations	Proposed pathways	Ref.
*Npr1*	CM specific KO	None	Not described/mice	Increase in metabolic processes	circRNA and microRNA	[47]
*Npr1*	CM specific KO	None	8 weeks/mice	Metabolic deregulation	Positive enrichment in nucleotide synthesis and histidine metabolism, negative enrichment of mitochondrial proteins	[48]
*Nppa*	Global KO	*Nppa*^−/−^ + I/R	8–10 weeks/mice	Increased infarct size and reduced autophagy	ANP/NPR1/PRKG-mediated stimulation of autophagy through activation of TFEB	[38]
ANP	Embryonic ventricular cell cultures	ANP treatment	E11.5/mice	Increased VCS cell proliferation	ANP/NPRA-mediated increase in PPARG and FAO promotes VCS formation	[49]
ANP	Primary cardiomyocyte cultures	ANP treatment	Adult/rats	Reduced cardiomyocyte apoptosis	ANP- and CNP-mediated DrpI phosphorylation and caspase 9 decrease	[50]
CNP		CNP treatment				
BNP	No genetic modification	4 weeks of BNP administration 7 days after STZ-induced diabetic cardiomyopathy	8 weeks/mice	Preservation of mitochondrial function and prevention of DMC onset	BNP/NPRA/PKG/STAT3-OPA1-mediated mitochondrial fusion activation	[51]
		AAV9-shBNP after 5 days of STZ-induced diabetic cardiomyopathy		Impaired mitochondrial function, exaggerated cardiac dysfunction		
Electrophysiology						
NPs or NP receptors	Experimental model	Manipulation	Age/species	Intracardiac observations	Proposed pathways	Ref.
*Npr2*	Global KO (*Npr2^−/+^*)	none	20 weeks/mice	Increased cSNRT, spontaneous AP frequency, slower HR, reduced If and Ica, L currents	NPR-B/cGMP-mediated inhibition of PDE3	[52]
*Nppa*, *Nppb*	Global KO *Nppa*^−/−^ or *Nppb^−/−^*	none	1–12 months/mice	Mild ventricular remodeling, no cardiac functional dysfunction	ANP, BNP/cGMP/PKG1/p38MAPK-mediated phophorylation of CREB	[53]
		TAC				
		Isoproterenol treatment		Increased incidence of ventricular arrythmias		
*Npr3*	Global KO	None	10–15 weeks/mice	SAN dysfunction, atrial suspeptibility and increased fibrosis	N/A	[54]
*Npr3*	Global KO	none	10–15 weeks/mice	SAN dysfunction and increased SAN fibrosis	N/A	[55]
		Ang-II treatment				
*Npr3*	Global KO	none	10–15 weeks/mice	Atrial suspeptibility and increased atrial fibrosis	N/A	[56]
		Ang-II treatment				
Contractility						
NPs or NP receptors	Experimental model	Manipulation	Age/species	Intracardiac observations	Proposed pathways	Ref.
*Npr2*	CM specific *Npr2* KO	TAC	2 months/mice	Increased ventricular stiffness, LV diastolic and systolic dysfunction	CNP/NPRB/cGMP/PKGI-mediated Ser4080 phosphorylationof Tintin	[57]
CNP, BNP	cGMP biosensor	Isolated cardiomyocytes	Neonatal-adult/rats	Increased lucitropic and negative inotropic effects by CNP	CNP increases cGMP near TnI and PLB, regulated by PDE2 and PDE3 BNP increases cGMP only near PLB	[58]

## Data Availability

Not applicable.

## Abbreviation

ANP	Atrial natriuretic peptide
ANF	Atrial natriuretic factor
BNP	B-type/Brain natriuretic peptide
CNP	C-type natriuretic peptide
NPR-A	Natriuretic peptide receptor-A
NRP-B	Natriuretic peptide receptor-B
NRP-C	Natriuretic peptide receptor-C
*Nppa*	Natriuretic peptide A
*Nppb*	Natriuretic peptide B
*Nppc*	Natriuretic peptide C
*Npr1*	Natriuretic peptide receptor-1
*Npr2*	Natriuretic peptide receptor-2
*Npr3*	Natriuretic peptide receptor-3
